# Independent and sex-stratified association between microsatellite instability and peripheral hemoglobin in colorectal cancer

**DOI:** 10.3389/fonc.2026.1890542

**Published:** 2026-07-15

**Authors:** Yang Lu, Hao Wang, Gang Chen

**Affiliations:** 1 Department of General Surgery, Nanjing Drum Tower Hospital Clinical College of Nanjing Medical University, Nanjing, Jiangsu, China; 2 Department of General Surgery, Nanjing Drum Tower Hospital, The Affiliated Hospital of Nanjing University Medical School, Nanjing, Jiangsu, China

**Keywords:** anemia, biomarker, colorectal cancer, hemoglobin, microsatellite instability, screening, tumor microenvironment

## Abstract

**Background:**

Microsatellite instability (MSI) informs colorectal cancer (CRC) prognosis and treatment. Although anemia is common in CRC, whether peripheral hemoglobin (HB) differs according to MSI status independently of sex, tumor location, stage, and selected nutritional covariates remains unclear. This exploratory study evaluated the association between MSI status and HB and described sex-stratified discrimination within the study cohort.

**Methods:**

This retrospective study included 400 patients with stage II-III CRC. MSI-H status was inferred from four-protein mismatch repair immunohistochemistry. Multivariable logistic regression assessed HB as a continuous predictor after adjustment for sex, age, tumor location, TNM stage, albumin, body mass index, log-transformed C-reactive protein, hypertension, and diabetes. Sex-stratified receiver operating characteristic analyses evaluated HB alone and an expanded model, with 1, 000 bootstrap resamples for internal validation.

**Results:**

The cohort included 95 MSI-H and 305 microsatellite-stable patients. HB was lower in MSI-H patients of both sexes (both P < 0.001). Higher HB remained inversely associated with MSI-H status after adjustment (OR per 1-g/L increase = 0.977, 95% CI: 0.966-0.988; P < 0.001). The cohort-derived HB cutoffs were 125.0 g/L for males (AUC = 0.654) and 98.0 g/L for females (AUC = 0.693). Expanded-model apparent AUCs were 0.740 and 0.762, while bootstrap-corrected AUCs were 0.678 and 0.700, respectively.

**Conclusions:**

Lower HB was independently associated with MSI-H status, but its discrimination was modest and the expanded models showed optimism. HB may warrant further evaluation as an adjunctive signal, but it cannot replace tumor-based MMR/MSI testing. Prospective external validation is required.

## 1 Introduction

Colorectal cancer (CRC) is a biologically heterogeneous disease regulated by multiple molecular pathways, including noncoding RNA-mediated networks that influence tumor-cell survival and metastasis (1). Microsatellite instability (MSI) is an established molecular subtype relevant to prognostication and treatment selection in CRC (2). Recent computational and image-based approaches have further improved MSI prediction and tumor risk stratification in digestive tract cancers (3). MSI-high (MSI-H) tumors are characterized by high tumor mutational burden and a distinct immune microenvironment (4). Anemia is also common in CRC and may reflect gastrointestinal blood loss, nutritional deficiency, chronic inflammation, comorbidity, or advanced disease (5, 6). However, comparatively little is known about whether routine peripheral HB varies with the MSI phenotype after accounting for major clinicopathological factors.

The specific contribution of this study is therefore not to establish anemia as a new cancer phenomenon, but to test whether HB is associated with a defined molecular subtype of CRC and whether this association persists within sex strata and after expanded adjustment for demographic, clinicopathological, nutritional, inflammatory, and comorbidity-related covariates. We analyzed 400 patients with non-metastatic stage II-III CRC and explored sex-specific HB thresholds as descriptive, cohort-derived markers. Given the retrospective design and absence of external validation, these thresholds were considered hypothesis-generating rather than ready for clinical implementation.

## 2 Materials and methods

### 2.1 Study population

We retrospectively analyzed 400 patients with stage II-III CRC who underwent radical resection at Nanjing Drum Tower Hospital from May 2022 to June 2025. Inclusion criteria required complete preoperative abdominopelvic CT imaging, peripheral blood testing within 30 days before surgery, and clearly documented MMR status. Preoperative HB, serum albumin, CRP, height, and weight were measured within this 30-day window; BMI was calculated as weight divided by height squared. Hypertension and diabetes were obtained from the recorded medical history. Patients with concurrent malignancies or prior neoadjuvant therapy were excluded. Detailed iron indices, renal function, medication exposure, and a broader structured comorbidity profile were not consistently available.

### 2.2 MSI determination and statistical analysis

Mismatch repair status was abstracted from pathology reports based on immunohistochemical assessment of all four proteins (MLH1, PMS2, MSH2, and MSH6). Deficient mismatch repair (dMMR), defined as loss of nuclear expression of at least one protein with retained internal controls, was used as a surrogate for the MSI-H phenotype (7). Cases without a clearly documented MMR classification were not included. The retrospective dataset contained the final IHC classification rather than individual staining patterns, and paired PCR- or next-generation sequencing-based MSI confirmation was not systematically available; therefore, ambiguous staining and IHC-molecular discordance could not be independently adjudicated. Analyses were performed using R software (version 4.5.2), and the revised analyses and figure were independently reproduced using Python 3.10.8. Approximately normally distributed continuous variables were summarized as mean (standard deviation) and compared using independent-samples t tests; CRP was summarized as median (interquartile range) and compared using the Mann-Whitney U test because of marked right skew. Categorical variables were compared using Pearson’s chi-square test with Yates’ continuity correction for 2 x 2 tables. Variables were analyzed on their original scales, except CRP, which was entered as ln(CRP + 1). For the primary multivariable logistic regression, MSI-H status was the binary outcome and HB was entered as a continuous predictor; the HB odds ratio therefore represents the change in odds of MSI-H associated with each 1-g/L increase in HB. The expanded model adjusted for sex, age, tumor location (left vs. right), TNM stage (III vs. II), serum albumin, BMI, ln(CRP + 1), hypertension, and diabetes. Sex, hypertension, diabetes, tumor location, and TNM stage were entered as categorical variables; the remaining predictors were continuous. All 400 patients had complete data for these variables. Sex-stratified ROC curves were then used to describe discrimination by HB alone and by the expanded model including HB, age, albumin, BMI, ln(CRP + 1), hypertension, diabetes, tumor location, and TNM stage; sex was not re-entered into the sex-stratified models. Cutoffs were selected using Youden’s index. Internal validation of each sex-stratified expanded model used 1, 000 bootstrap resamples with a fixed random seed (20260615). In each resample, model optimism was calculated as the difference between the AUC in the bootstrap sample and the AUC obtained when that bootstrap-fitted model was applied to the original subgroup; mean optimism was subtracted from the apparent AUC. Bootstrap distributions of the HB cutoffs were also summarized using the 2.5th and 97.5th percentiles. No external validation was performed. All tests were two-sided, and P < 0.05 was considered statistically significant.

## 3 Results

### 3.1 Patient characteristics and multivariate analysis

The cohort included 305 MSS (76.2%) and 95 MSI-H (23.8%) patients (**Table 1**). Female patients were more frequent in the MSI-H group (48.4% vs. 34.8%, P = 0.023). In sex-stratified comparisons, MSI-H patients had lower HB among both males and females (both P < 0.001). In the expanded multivariable model adjusted for sex, age, tumor location, TNM stage, serum albumin, BMI, ln(CRP + 1), hypertension, and diabetes, higher HB remained inversely associated with MSI-H status (OR per 1-g/L increase = 0.977, 95% CI: 0.966-0.988; P < 0.001), corresponding to approximately 2% lower odds per 1-g/L increase within this model. Sex was not independently associated after adjustment (OR = 1.34, 95% CI: 0.80-2.25; P = 0.263). These findings describe an adjusted association, not causality or diagnostic performance.

**Table 1:** Baseline characteristics and multivariate logistic regression for MSI-H status.

Variables	MSS (n=305)	MSI-H (n=95)	Univariate P value	Adjusted OR (95% CI)^a^	P value
Sex [n (%)]			**0.023**	1.34 (0.80-2.25)	0.263
Male	199 (65.2%)	49 (51.6%)			
Female	106 (34.8%)	46 (48.4%)			
Age (years)	62.76 ± 9.78	58.16 ± 12.69	**<0.001**	**0.95 (0.93-0.98)**	**<0.001**
Hemoglobin (g/L)				**0.98 (0.97-0.99)**	**<0.001**
Male subgroup	127.5 ± 25.3	113.4 ± 28.1	**<0.001**		
Female subgroup	115.3 ± 19.3	101.7 ± 20.2	**<0.001**		
Albumin (g/L)	39.78 ± 4.36	38.60 ± 3.61	**0.017**	0.99 (0.92-1.06)	0.758
BMI (kg/m²)	23.56 ± 3.15	23.48 ± 3.89	0.836	0.98 (0.91-1.06)	0.684
CRP (mg/L), median (IQR)	5.4 (3.2-10.3)	7.2 (4.0-19.5)	0.020	1.27 (0.95-1.70)^b^	0.110
Hypertension [n (%)]	99 (32.5%)	27 (28.4%)	0.540	0.97 (0.54-1.73)	0.911
Diabetes [n (%)]	59 (19.3%)	18 (18.9%)	1.000	1.08 (0.57-2.05)	0.803
TNM stage III [n (%)]	110 (36.1%)	24 (25.3%)	0.068	0.59 (0.34-1.05)	0.071
Left-sided tumor [n (%)]	94 (30.8%)	22 (23.2%)	0.191	0.93 (0.51-1.68)	0.798

Bold values indicate statistically significant P values (P < 0.05).

^a^ The expanded model included sex, age, tumor location, TNM stage, albumin, BMI, ln(CRP + 1), hypertension, diabetes, and HB.

^b^ OR for CRP is reported per 1-unit increase in ln(CRP + 1).

### 3.2 Sex-Stratified and multivariate ROC analyses

**Figure 1:**
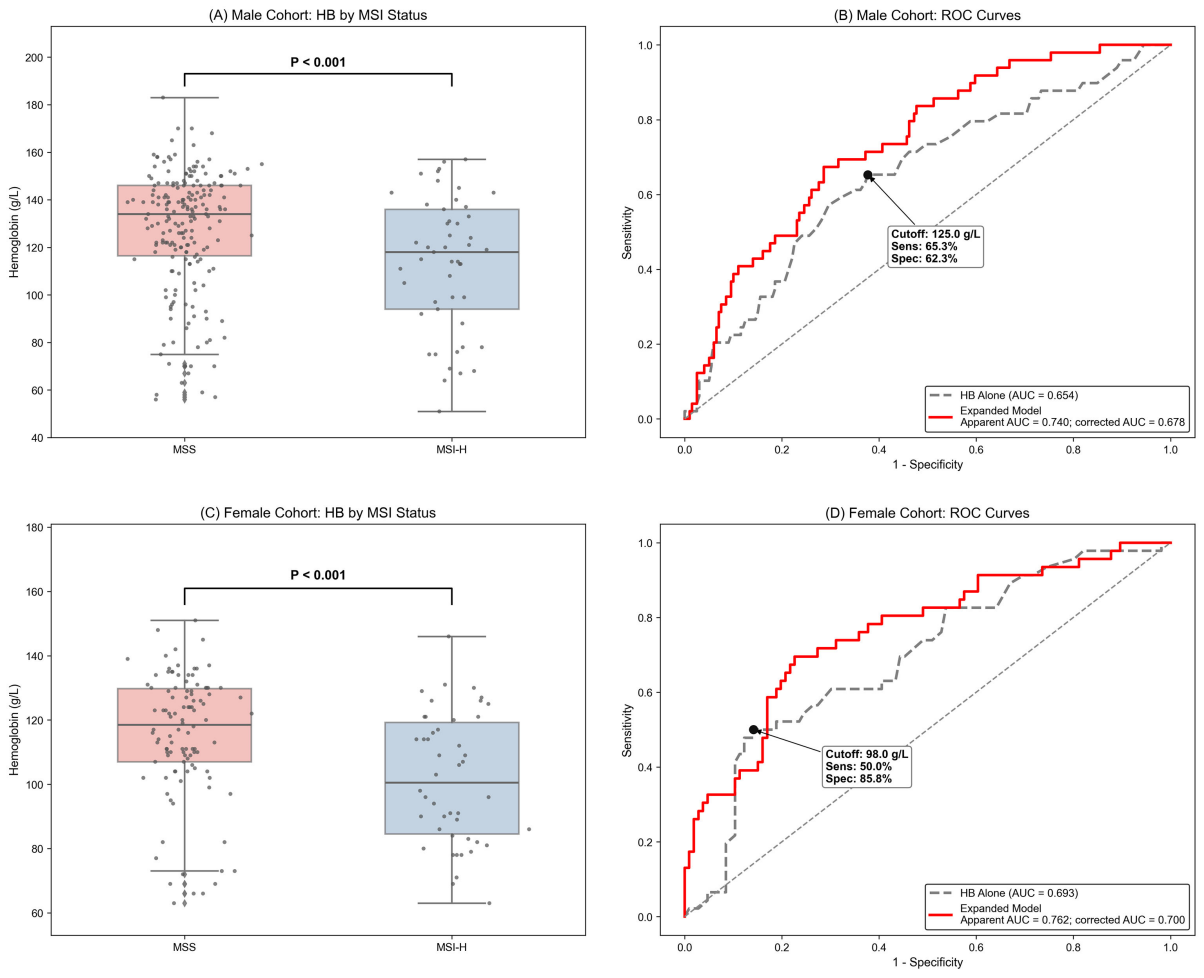
Peripheral hemoglobin levels and sex-stratified exploratory ROC models for MSI-H status. **(A, C)** Boxplots with swarm plots showing peripheral hemoglobin (HB) levels in the MSS and MSI-H groups among males **(A)** and females **(C)**, with P values from independent-samples t tests. **(B, D)** ROC curves showing the apparent performance of HB alone (dashed lines) and an expanded multivariable model including HB, age, serum albumin, BMI, ln(CRP + 1), hypertension, diabetes, tumor location, and TNM stage (solid lines) in male **(B)** and female **(D)** subgroups. Solid circles indicate cohort-derived cutoffs for HB alone (125.0 g/L for males and 98.0 g/L for females), selected using Youden’s index. The ROC curves display apparent derivation-cohort performance. The figure legends also report AUCs after 1, 000-resample bootstrap optimism correction (0.678 for males and 0.700 for females). No external validation was performed.

Sex-stratified ROC analyses described the ability of peripheral HB alone and the expanded multivariable model to distinguish MSI-H from MSS within the derivation cohort (**Figure 1**). For males, the data-derived HB cutoff was 125.0 g/L, with an AUC of 0.654, sensitivity of 65.3%, and specificity of 62.3%. For females, the cutoff was 98.0 g/L, with an AUC of 0.693, sensitivity of 50.0%, and specificity of 85.8%. The expanded model yielded apparent AUCs of 0.740 for males and 0.762 for females. After 1, 000 bootstrap resamples, mean optimism was 0.061 in males and 0.062 in females, producing optimism-corrected AUCs of 0.678 and 0.700, respectively. The bootstrap distributions of the HB cutoff were broad: 113–138 g/L in males and 91–121 g/L in females (2.5th-97.5th percentiles). These findings indicate modest discrimination and uncertainty in the cohort-derived thresholds. The models and cutoffs have not undergone external validation and should not be interpreted as established clinical accuracy.

## 4 Discussion

In this retrospective cohort, lower peripheral HB was independently associated with MSI-H status after expanded adjustment for sex, age, tumor location, TNM stage, serum albumin, BMI, ln(CRP + 1), hypertension, and diabetes. The novelty of this finding lies in linking a routine hematological measure to a defined molecular subtype rather than reiterating the general association between anemia and CRC. The persistence of the association within both sex strata and after clinicopathological adjustment suggests that the finding is not explained solely by the unequal sex distribution or right-sided tumor location. Nevertheless, residual confounding remains likely, and the observational analysis cannot establish that MSI-H biology causes lower HB.

One possible explanation is that the immune-rich MSI-H microenvironment may contribute to systemic inflammatory signaling, altered iron handling, and impaired erythropoiesis through pathways that could include cytokines and hepcidin (8, 9). This interpretation is a hypothesis only. Alterations in immune and inflammatory microenvironments also influence progression in other chronic diseases, illustrating the broader role of systemic inflammation in human pathology (10). Although CRP was included as an available systemic inflammatory marker, we did not measure hepcidin, iron indices, or specific inflammatory cytokines, and other common causes of anemia in CRC, including occult blood loss, nutritional deficiency, renal dysfunction, and comorbidity, may also contribute. Mechanistic studies with direct biomarker measurements are required before a cytokine-hepcidin pathway can be inferred.

The ROC results also require a cautious interpretation. HB alone showed modest discrimination (AUC 0.654 in males and 0.693 in females), while the expanded models showed moderate discrimination (apparent AUC 0.740 and 0.762, respectively). Bootstrap correction reduced the AUCs to 0.678 and 0.700, indicating meaningful optimism and reinforcing the exploratory nature of the models. Recent multi-omics and pathology-integrated approaches have improved survival risk stratification across cancers, supporting the general value of biomarker-based models while also illustrating the need for rigorous validation (11). The female cutoff had relatively high specificity but low sensitivity, and bootstrap resampling showed broad cutoff distributions in both sexes. In CRC practice, preoperative decisions commonly integrate multiple clinical risk factors and diagnostic modalities (12); a single routine blood measure should therefore be viewed only as a possible adjunct. Accordingly, HB should not be used to diagnose MSI-H or replace guideline-recommended tumor-based MMR/MSI testing. A potential future role, if prospectively validated, would be as an adjunctive prompt in settings where molecular results are delayed or unavailable: an unexpectedly low sex-specific HB could remind clinicians to confirm that appropriate MMR/MSI testing has been completed. Whether this approach improves testing completion, timeliness, or cost-effectiveness must be evaluated directly before incorporation into clinical workflows.

This study has several limitations. First, its retrospective, single-center design introduces the potential for selection bias and limits generalizability. Although 1, 000-resample bootstrap validation quantified and corrected model optimism, no independent external validation was available. The corrected AUCs were lower than the apparent values, and the broad bootstrap cutoff distributions indicate that the models and thresholds remain exploratory. Moreover, the sex-stratified expanded models included nine predictors but only 49 male and 46 female MSI-H events, so overfitting remains possible despite bootstrap correction. Second, the expanded model incorporated CRP, BMI, hypertension, and diabetes in addition to albumin and clinicopathological variables, but important determinants of HB remained unavailable or incompletely recorded, including ferritin, transferrin saturation, serum iron, renal function, detailed comorbidity burden, medication use, peri-tumoral bleeding, and broader nutritional measures. Thus, residual confounding cannot be excluded. Third, dMMR by four-protein IHC was used as a surrogate for MSI-H. Paired molecular MSI testing and individual staining data were not systematically available, so ambiguous staining and potential IHC-molecular discordance could not be independently reviewed. Fourth, the proposed cytokine-hepcidin mechanism was not directly tested. Finally, the modest AUCs and limited sensitivity, particularly in females, preclude immediate clinical application. Prospective, multicenter studies with prespecified models, complete anemia-related covariates, and independent validation are needed.

## 5 Conclusions

Lower peripheral HB was independently associated with MSI-H status in this cohort of stage II-III CRC patients. Although discrimination was modest and bootstrap validation indicated limited incremental discrimination of the expanded model, HB may provide clinically relevant information associated with MSI status. As a readily available and low-cost parameter, HB could be further evaluated as one component of a multimodal triage strategy, but it should not replace tumor-based MMR/MSI testing. Prospective multicenter external validation is required to determine its clinical utility.

## Data Availability

The raw data supporting the conclusions of this article will be made available by the authors, without undue reservation.
